# The influence of bone quality on radiological outcome in 50 consecutive acetabular fractures treated with a pre-contoured anatomic suprapectineal plate

**DOI:** 10.1007/s00402-021-03867-3

**Published:** 2021-03-24

**Authors:** Maximilian J. Hartel, Tareq Naji, Florian Fensky, Frank O. Henes, Darius M. Thiesen, Wolfgang Lehmann, Karl-Heinz Frosch, Dimitris Ntalos

**Affiliations:** 1 Department of Trauma and Orthopaedic Surgery, University Medical Center Hamburg-Eppendorf, Martinistraße 52, 20246 Hamburg, Germany; 2 Department of Trauma, Orthopaedic Surgery and Sports Traumatology, BG Trauma Hospital Hamburg, Hamburg, Germany; 3 Radiologische Allianz, Hamburg, Germany; 4 Department of Diagnostic and Interventional Radiology and Nuclear Medicine, University Medical Center Hamburg-Eppendorf, Hamburg, Germany; 5 Department of Trauma Surgery, Orthopaedic Surgery and Plastic Surgery, University Medical Center Göttingen, Göttingen, Germany

**Keywords:** Acetabular fracture, Bone mass density, Suprapectineal plate, Anatomic plate, Geriatric traumatology

## Abstract

**Purpose:**

To investigate the range of indications of an anatomical-preshaped three-dimensional suprapectineal plate and to assess the impact of the bone mass density on radiologic outcomes in different types of acetabular fractures.

**Patients and methods:**

A consecutive case series of 50 acetabular fractures (patient age 69 ± 23 years) treated with suprapectineal anatomic plates were analyzed in a retrospective study. The analysis included: Mechanism of injury, fracture pattern, surgical approach, need for additional total hip arthroplasty, intra- or postoperative complications, as well as bone mass density and radiological outcome on postoperative computed tomography.

**Results:**

Most frequently, anterior column fracture patterns with and without hemitransverse components as well as associated two column fractures were encountered**.** The anterior intrapelvic approach (AIP) was used in 98% (49/50) of the cases as primary approach with additional utilization of the first window of the ilioinguinal approach in 13/50 cases (26%). Determination of bone density revealed impaired bone quality in 70% (31/44). Postoperative steps and gaps were significantly greater in this subgroup (*p* < 0.05). Fracture reduction quality for postoperative steps revealed anatomic results in 92% if the bone quality was normal and in 46% if impaired (*p* < 0.05). In seven cases (14%), the plate was utilized in combination with acute primary arthroplasty.

**Conclusion:**

A preshaped suprapectineal plate provides good radiological outcomes in a variety of indications in a predominantly geriatric cohort. Impaired bone quality has a significantly higher risk of poor reduction results**.** In cases with extensive joint destruction, the combination with total hip arthroplasty was a valuable option.

## Introduction

In recent years, acetabular fractures have shown an increasing incidence, in the elderly. As opposed to younger individuals, they are commonly the result of a low-impact trauma and lead to poorer radiological and clinical outcome. Anatomical reduction and its retention are the main goals of the surgical treatment of unstable acetabular fractures [1–5]. Several poor prognostic factors for open reduction and internal fixation (ORIF) in the elderly have been described such as posterior wall comminution, marginal impaction, femoral head impaction or hip dislocation. Total hip arthroplasty (THA) provides an additional treatment option in elderly patients with the rate of conversion to THA following ORIF reported to be approximately 20–25% [2–4, 6, 7]. During ORIF procedures, traditionally, access is established via the ilioinguinal- or Kocher–Langenbeck approach to either the anterior or the posterior fracture patterns. Less invasive anterior approaches, such as the anterior intrapelvic approach (AIP) are increasingly used [8–14]. Well-established fracture fixation methods include various combinations of pelvic reconstruction plates and lag screws. Newer buttress plate constructs, which cover the posterior and anterior columns through the quadrilateral surface, have been developed to take advantage of the exposure of both columns provided by the AIP [13–16]. Providing medial buttressing to the posterior column and quadrilateral surface may be beneficial in terms of stability and prevention of medial subluxation when compared with traditional fracture fixation techniques [15, 16]. While the conventional plates are either shaped in a straight or curved fashion, more recently buttress plates were introduced as three-dimensional shaped plates to fit anatomically to the periacetabular region [9]. In other anatomical regions, the use of anatomically pre-contoured plates is already well established, while in the context of acetabular fractures, only one study has shown promising results so far [9, 17].

This retrospective study was conducted to identify and describe the indications and radiological outcomes following the use of an anatomic suprapectineal plate. Furthermore, in the setting of a foremost geriatric patient collective, the influence of bone mass density on radiological outcomes was analyzed and the additional necessity of THA was determined.

## Methods

A consecutive case series of 50 acetabular fractures treated with suprapectineal plates (Pelvic Pro; Stryker GmbH, Selzach, Switzerland) in a single, level one trauma center was analyzed. All methods were conducted in accordance with relevant guidelines and regulations. All experimental protocols were approved by and informed consent was obtained from all subjects according to the Ethics Committee of the University Medical Center Hamburg-Eppendorf (Hamburg Medical Chamber, Hamburg, study number: PF4812).

The operating procedure was performed in a standardized manner by three experienced pelvic surgeons. The institutions standard anterior approach is the anterior intrapelvic approach (also known as the modified Stoppa approach) [9, 18, 19]. After fracture reduction and its temporary retention with reduction clamps, K-wires and lag screws, the anatomical-preshaped suprapectineal plate was positioned and fixated with screws. Where needed, the AIP approach was mainly combined with not only the first ilioinguinal window, but also different other approaches (in this series: Smith–Peterson approach and Kocher–Langenbeck approach). If the fracture was considered to be non-reconstructible an additional total hip arthroplasty was performed.

The retrospective analysis included: Mechanism of injury, fracture patterns according to Letournel [20], surgical approach, need for total hip arthroplasty and intra- or postoperative complications. The fracture reduction quality (gaps and steps) was measured at any location in the joint (weight- and non-weight-bearing areas) by a 5th year senior resident doctor specializing in radiology (TN) on postoperative computed tomographies (CT) scans according to Matta radiographic grading (anatomic: 0–1 mm, imperfect: 2–3 mm and poor: > 3 mm) was used for interpretation [21]. Furthermore, the bone density was measured using CT scans and recorded in Hounsfield units of the vertebral body of L5. The assessment was performed, as previously described, by placing a single oval over the trabecular bone in the axial view. In the sagittal plane, the correct positioning in the middle of the vertebral body was verified [22, 23]. As previously described, bone mass density of less than 150 HU was defined as impaired bone quality (osteoporotic and osteopenic) and above 150 HU as normal [22, 23]. Overall, 44 of 50 CT scans could be identified that allowed for the bone quality analysis and 38 CT scans for analysis of fracture reduction quality. Five cases that received an acute total hip and one case of a 96-year-old lady, who deceased before the postoperative CT scan were excluded from the fracture reduction quality analysis. All fracture reduction quality cases were available for bone quality analysis and subcategorized accordingly as described above in normal (*n* = 12) or impaired bone quality (*n* = 26). The presence of marginal impaction was noted in 16 cases. All of these had reduced bone quality. Cases of doubt were furthermore reviewed in an interdisciplinary consensus meeting with one board-certified orthopedic trauma surgeon and one senior orthopedic trauma surgeon specializing in pelvic and acetabular surgery.

### Statistical analysis

For descriptive statistical analysis, mean values and ranges were ascertained for all measurements. Where appropriate, significance was tested by Mann–Whitney *U* test and Pearson’s Chi-squared test with a *p* value of < 0.05 indicating statistical significance. A scatter plot was used to further depict the interrelationship between reduction results and Hounsfield unit measurements.

## Results

Patient age averaged 69 ± 18 years (range 23–96 years) and the study group consisted of 74% male and 26% female patients, with a male-to-female ratio of 3:1. Determination of bone density revealed impaired bone quality in 70% (31/44) of the cases. In this cohort, the mean age was 77 ± 9 years (range 58–96 years) compared to a mean age of 46 ± 19 years (range 23–86 years) if the bone quality was normal (13/44; 30%).

20% (10/50) of the patients showed a simple pattern and 80% (40/50) an associated pattern according to Letournel’s classification. Most frequently, anterior column fracture patterns with and without hemitransverse components as well as associated both column fractures were encountered (Table 1). The most frequent cause of injury were falls (34/50, 68%), followed by motor vehicle accidents (7/50, 14%; Table 1).

**Table 1:** Absolute and relative distribution of injury mechanisms and associated fracture patterns according to the Letournel classification

Mechanism of injury	Absolute and relative distribution
Fall < 3 m	27 (54%)
Fall > 3 m	7 (14%)
Motor vehicle accident	7 (14%)
Bicycle accident	6 (12%)
Ski accident	1 (2%)
Non-traumatic	2 (4%)
Polytrauma	17 (34%)
Fracture pattern simple (Letournel)	
Posterior wall	0 (0%)
Posterior column	0 (0%)
Anterior wall	0 (0%)
Anterior column	8 (16%)
Transverse	2 (4%)
Fracture pattern associated (Letournel)	
Posterior column + posterior wall	1 (2%)
Transverse + posterior wall	0 (0%)
T-style	5 (10%)
Anterior column + posterior hemitransverse	18 (36%)
Both columns	16 (32%)

**Fig. 1:**
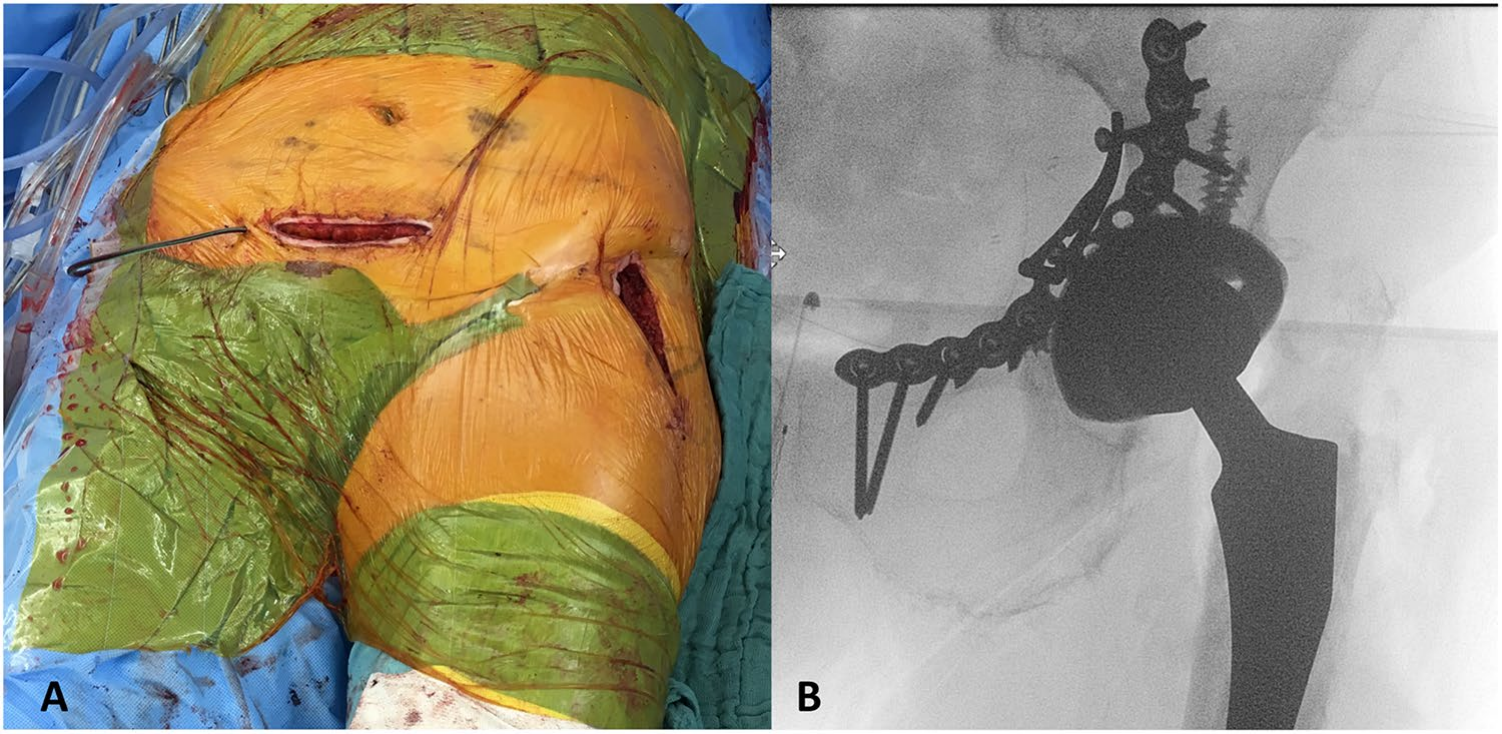
**a** Intraoperative picture of the AIP and DAA approaches combined, and **b** corresponding final intraoperative radiograph after finished acute total hip arthroplasty

The AIP approach was used in 98% (49/50) of the cases as primary approach. In 13 cases (26%), the first window of the ilioinguinal approach was additionally used. In one case, the AIP approach was first enlarged by the first and for even better visualization by the second window, as well. In three cases, an additional Kocher–Langenbeck (KL) approach was performed to address the posterior column and wall. In one patient, the AIP and KL approaches were utilized simultaneously in a floppy lateral patient positioning technique. In another case, a THA was performed after double-plating of the posterior column and wall in a lateral position. The direct anterior approach (DAA) utilizing the Smith Petersen interval was additionally used in the following four cases: In three periprosthetic fracture cases, plate osteosynthesis was performed from the inside through the AIP and revision arthroplasty was performed from outside using the DAA. Figure 1 shows an example of this technique.

Finally, one younger (23 years) patient with normal bone quality had an additional fracture pattern at the anterior wall, which was addressed using the DAA approach, as well. One case had multiple prior urologic operations at the ventral aspect of the pelvis, which is why, in this exception, a pararectus approach as described by Marius Keel was chosen as primary approach (Table 2) [12].

**Table 2:** Overview of the different surgical approaches used in the cohort

Surgical approach	Absolute and relative distribution
AIP	49 (98%)
Pararectus	1 (2%)
Additional surgical approach (21/50, 42%)	
1st ilioinguinal window 1st and 2nd ilioinguinal window	13 (26%) 1 (2%)
Kocher–Langenbeck	3 (6%)
DAA/Smith Petersen	4 (8%)

**Fig. 2:**
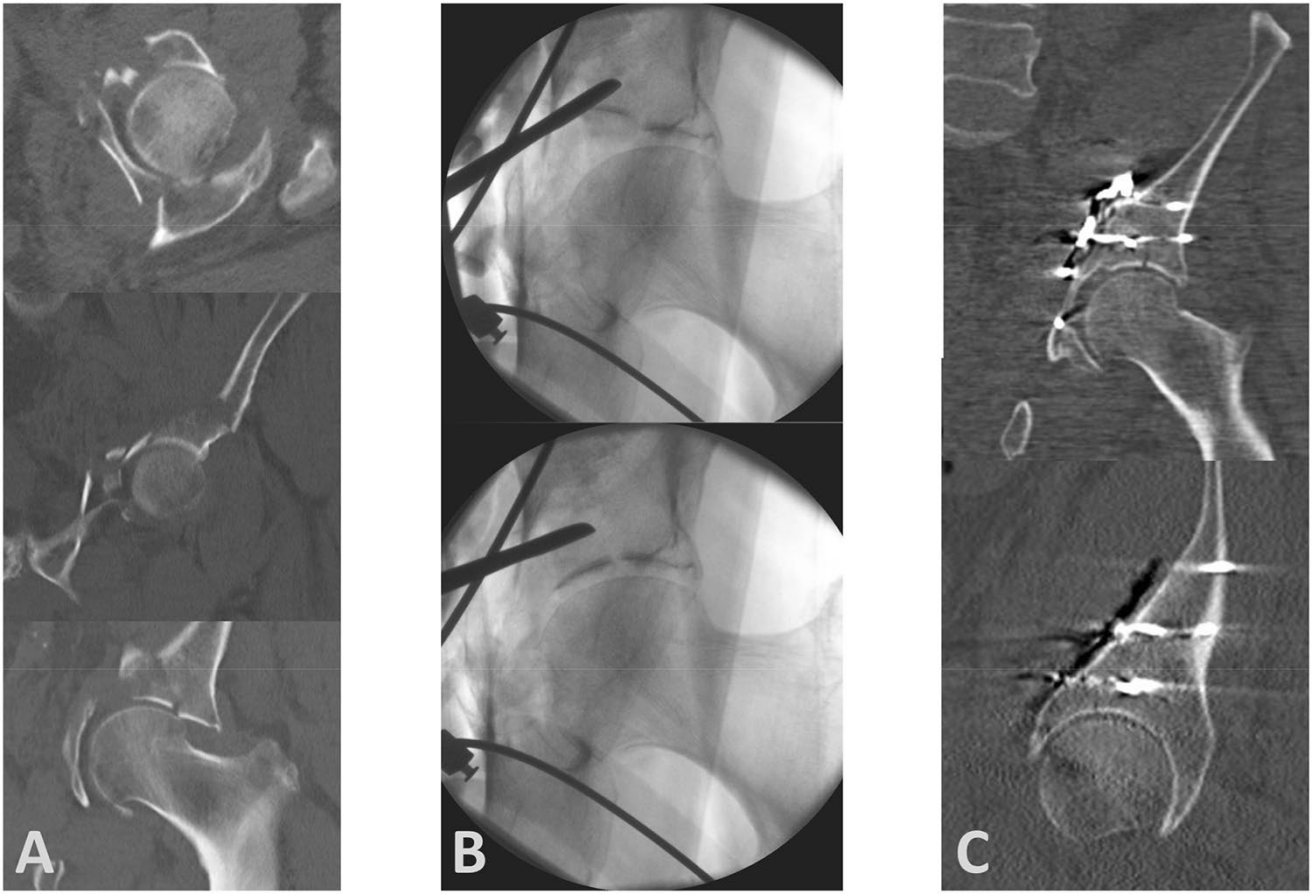
Example of an anterior column type acetabular fracture in a 72-year-old male with impaired bone quality and a large marginal impaction (gull sign). **a** Preoperative CT scan displaying a comminuted and impacted joint. **b** Intraoperative fluoroscopic images showing the head reduced from its subluxation using axial traction (above) and a reduction maneuver of the impacted joint surface using a rasp elevator. **c** Postoperative CT with a very successful yet imperfect reduction result (gap: 2 mm, step: 0 mm)

Fracture steps and gaps were classified according to Matta and revealed anatomic reduction results in 23/38 (61%), imperfect results in 11/38 (29%) and poor results in 4/38 (10%) for postoperative steps and 26/38 (69%) anatomic, 7/38 (18%) imperfect and 5/38 (13%) poor results for postoperative gaps. 12 out of these 38 patients showed normal bone quality, whereas 26 patients showed impaired bone quality. Analyzing potential influence of bone mass density on radiological outcome revealed anatomic results in 11/12 (92%, steps) and 9/12 (75%, gaps) cases if the bone quality was normal compared to 12/26 (46%, steps) and 17/26 (65%, gaps) if the bone quality was impaired (*p* < 0.05 for steps and *p* = 0.55 for gaps). Marginal impaction was detected in 16 of the 26 cases with impaired bone quality. Figure 2 shows an example of a 72-year-old male with impaired bone quality and a large marginal impaction (gull sign) reduced almost anatomically.

**Fig. 3:**
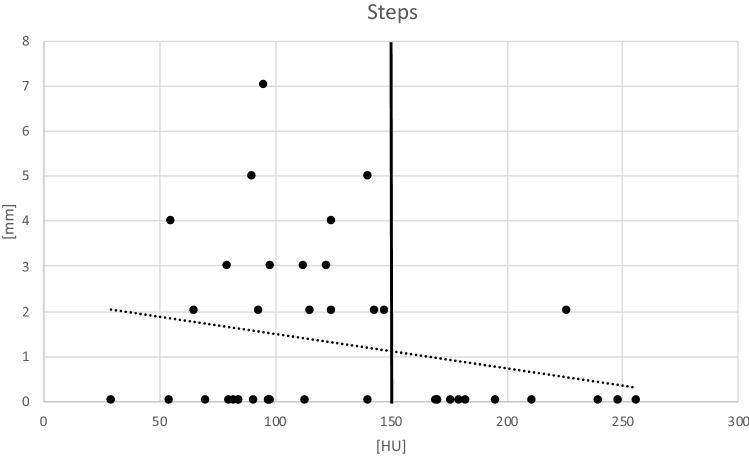
The remaining postoperative steps compared to the bone density estimated by the HU measurements

The mean overall postoperative step at any location in the joint (weight and non-weight bearing areas) was 1.2 ± 1.8 mm and the mean postoperative gap 1.2 ± 1.7 mm. A subgroup analysis showed that in cases with impaired bone quality the steps and gaps encountered were significantly greater compared to patients with a normal bone density (steps: 1.7 ± 1.9 mm vs 0.2 ± 0.6 mm, *p* < 0.05; gaps: 1.3 ± 1.8 mm vs 1.0 ± 1.8 mm, *p* < 0.05). Figure 3 shows the data distribution of this analysis. In these, a more marked scattering can be observed in the range of cases below a bone quality of 150 HU’s. In the subgroup deemed to have a normal bone status, there is one outlier with a HU value of 226. This patient was a 28 years old, heavily obese, high-velocity polytrauma with a comminuted T-type fracture with ipsilateral SI joint involvement. In this case, anatomic reduction could not be obtained.

The presence of marginal impaction led to unsignificantly increased steps and gaps in patients with impaired bone quality (steps: 1.8 ± 1.5 mm vs 1.6 ± 1.6 mm; gaps 1.4 ± 1.5 mm vs 1.0 ± 2.1 mm; *p* = 0.5).

In this case series, a total of seven patients (14%, mean age 78 ± 8 years) were treated with total hip arthroplasty (THA). Five of the seven patients showed impaired bone quality. One additional case with impaired bone qualified for THA but the 96-year old’s condition deteriorated postoperatively and the patient deceased, before further procedures could be undertaken. In one patient with a severe dome impaction in a pathologic fracture, a suprapectineal plate was used for medial buttressing and combined with a posterior column plate and acute total hip arthroplasty via the Kocher–Langenbeck approach.

The analysis of the complications revealed one patient with an intraoperative injury to the internal iliac vein, which required primary repair and blood transfusions. Otherwise, no intraoperative complications occurred. Postoperative complications included urinary tract infection (6/50), pneumonia (4/50), stroke (1/50), anemia requiring blood transfusions (2/50), acute kidney failure (1/50), pulmonary embolism (1/50) and superficial wound complications (3/50), that could be managed non-operatively. The majority of the patients did not show any postoperative complications (35/50, 70%).

## Discussion

This study demonstrates the successful use of suprapectineal plates in a variety of indications in a cohort with a high percentage of patients with impaired bone quality. Moreover, bone quality was found to be a significant influencing factor on the reduction quality in this cohort.

Acetabular fracture reduction and its retention is a challenging procedure. To avoid fracture displacement, neutralizing fixation plates need to be as congruent to the bone surface as possible [24]. While traditionally the final shape of the plates had to be contoured intraoperatively, this plate allows for a direct fit due to its pre-shaped anatomical design without recontouring in the majority of the cases [9, 25]. Based on the radiological outcome of this study, the fracture reduction quality is anatomic in the majority of the cases (61–69%). This is comparable to other studies using traditional fixation methods and reporting a reduction quality < 2 mm in 40–84% of the patients [14, 26–28]. Still, the comparison is very limited due to different patient collectives (on average younger patients), different surgical approaches and measurement methods. Moreover, comparison is limited, as this paper reports on a patient subgroup, in which an anatomic suprapectineal plate was deemed necessary. Simpler fracture patterns, which did not require this plate, were treated the “traditional way” with lag screws and simple reconstruction plates.

In this study, impaired bone quality was identified as a significant influencing factor of the fracture reduction quality. While normal bone density was associated with foremost anatomic reduction quality (83%, steps and gaps combined), impaired bone quality increased the risk of worse reduction results. Therefore, respecting the bone quality might be of particular importance in an aging patient collective and may require different techniques including head-locking or buttress screws as well as bone void fillers or cement augmentation [3]. An age-related decrease in bone density results in a different mechanism of injury compared to acetabular fractures in young adults which are typically associated with high-energy trauma. Low-energy falls from a standing height are the most common reason for acetabular fractures in patients over 60 years of age which was the most common cause in this study collective as well. In the elderly, acetabular fractures typically show different patterns. Fractures of the anterior column, disruption of the quadrilateral surface as well as acetabular dome impaction following low-energy trauma [3, 6, 7, 29] are found predominantly. There are just a few studies focusing on radiological outcome in a geriatric patient collective. Miller et al. reported a poor reduction quality in half of the recorded patients (mean age of 67, range 59–82 years) and Matta demonstrated that the rate of anatomically reduced fractures decreased after the age of 40 [21, 30]. Similarly, Anglen et al. described less favorable results after open reduction and internal fixation in the elderly (range 61–88 years) especially when presenting with superomedial dome impaction. Among the described gull sign, comminution and extended fracture patterns have been reported to be risk factors for ORIF failure [3, 5]. Gras et al. used a similar pre-shaped plate compared to this study and revealed comparable radiological results (anatomic 40%, imperfect in 33% and poor in 27%) in a smaller but comparable patient collective with a mean age of 64 ± 8 years [9]. While ORIF is the most favorable choice of treatment in younger patients, the management of acetabular fractures in geriatric patients remains highly controversial. Alongside ORIF, percutaneous fixation and conservative treatment options, total hip arthroplasty is a promising treatment alternative [3, 31–33]. In this study, THA was used in 14% (7/50) of the patients if the fracture was considered to be non-reconstructible. The combination of ORIF and THA has been demonstrated to achieve good results in selected patients [34]. The goal of initial ORIF is the restoration of the acetabular columns to establish sufficient stability to allow press fit acetabular implant insertion. Especially in osteoporotic-associated fractures adequate bone stock is crucial for THA [3, 7, 35, 36]. Therefore, based on the result of this study, impaired bone quality might further be an important contributing factor in the decision which treatment option to choose. The methodology of estimating the bone quality as described in this paper may be used with any native CT scan [22, 23].

The limitations of the study include its retrospective nature and the relatively small number of patients as well as the selection of a subgroup, which was treated with a suprapectineal plate. Therefore, simpler fracture patterns were more likely excluded from the cohort, as they were treated with “traditional” reconstruction plates. Furthermore, since no clinical outcome parameters were obtained, direct clinical translation is limited. Still, fracture reduction quality is widely known to be a strong parameter determining post-traumatic osteoarthritis and clinical outcome. Moreover, in this study, postoperative CT scans were analyzed which could recently be shown to be superior to radiography for detecting residual displacement after acetabular fracture fixation [37]. Second, a 5th year senior resident specializing in radiology dealing with imaging techniques in his daily work, performed the primary measurements. This detail ensured a high probability of objective, valid and precise results. A main finding in this study is the increased risk of worse reduction results in cases of reduced bone quality. With the data available, it was not possible to differentiate whether this fact is due to a worse fracture reduction in the first place or because of worse retention of the fragments due to the reduced bone quality resulting in loss of reduction postoperatively. Future research is needed to answer this open question. For the estimation of the bone quality in the individual cases, a methodology was once again used that proved to be valid several times before [22, 23] [38–40]. Recent studies suggest measurements of three different vertebrae, however. Furthermore, there is an ongoing scientific debate on the exact cutoff values for the differentiation between healthy, impaired and osteoporotic bone stock [41]. Data previously published by these authors, indicate that a 150 HU’s seem to be a valid cut-off value for the differentiation between healthy and impaired bone quality [23].

In conclusion, this study demonstrates that the use of a pre-contoured anatomic suprapectineal plate via the AIP provides good radiological outcomes in a variety of indications. Patients with impaired bone quality have a significantly higher risk for worse reduction results. In selected cases with extensive joint destruction, the combination with total hip arthroplasty was found to be useful.
